# Incidental Diagnosis of Occult Maternal Malignancy With Routine Noninvasive Prenatal Testing During Pregnancy

**DOI:** 10.7759/cureus.76331

**Published:** 2024-12-24

**Authors:** Eniola R Ibirogba, Erica Macdonald, Michael E Tsimis, Marcos Cordoba, Mili Thakur, Vivian C Romero

**Affiliations:** 1 Maternal Fetal Medicine, The Ohio State University Wexner Medical Center, Columbus, USA; 2 Prenatal Genetics, Michigan State University College of Human Medicine/Corewell Health, Grand Rapids, USA; 3 Maternal Fetal Medicine, Michigan State University College of Human Medicine/Corewell Health, Grand Rapids, USA; 4 Reproductive Endocrinology and Infertility, The Fertility Center, Grand Rapids, USA

**Keywords:** cell-free dna (cfdna), noninvasive prenatal testing (nipt), oncofertility, pregnancy in cancer, prenatal genetic screening

## Abstract

The noninvasive prenatal test (NIPT) for genetic screening has been adopted globally as an alternative to first-trimester and quad screening due to its high sensitivity and specificity. NIPT involves detecting and processing foreign fetal DNA in maternal circulation to screen for fetal aneuploidy. An incidental consequence of this process is the detection of foreign tumor cell DNA in maternal circulation in otherwise asymptomatic patients. Here, we present two such cases of maternal cancer that were initially suspected with routine NIPT during pregnancy. The first patient had an "atypical finding" on NIPT at 13 weeks, five days gestation. Diagnostic prenatal genetic testing with amniocentesis at 16 weeks gestation showed a normal fetal karyotype. Between 25 and 28 weeks gestation, she developed nonspecific symptoms and was ultimately diagnosed with stage IIIB intrahepatic cholangiocarcinoma. The second patient had a "non-reportable" NIPT at 11 weeks due to insufficient fetal DNA fraction. At 16 weeks gestation, NIPT was repeated and showed an "atypical finding of maternal origin". The patient was enrolled in the National Institute of Health Incidental Detection of Maternal Neoplasia Through Non-Invasive Cell-Free DNA Analysis (NIH IDENTIFY) study. She had a whole-body magnetic resonance imaging (MRI) that identified an adrenal mass. She was diagnosed with stage III high-grade adrenocortical adenocarcinoma. NIPT is not validated for cancer screening or diagnosis. Although the overall incidence of cancer diagnosed with NIPT in otherwise asymptomatic patients is extremely low, this finding presents unique ethical challenges. A multidisciplinary approach is recommended.

## Introduction

In the last decade, noninvasive prenatal testing (NIPT) was incorporated into clinical practice as a highly sensitive and specific alternative to multi-marker screening. The sensitivity (97%, 99%, and 95%) and specificity (99.7%, 99.9%, and 99.9%) for the most common fetal autosomal aneuploidies (21, 18, and 13, respectively) are comparable in both high- and low-risk populations [1]. Other common conditions NIPT can detect include sex chromosome aneuploidies, triploidy, microdeletion, and microduplication syndromes [2,3].

Circulating cell-free DNA from maternal plasma is predominantly derived from apoptotic white blood cells. In certain instances, such as mosaicism, pregnancy, or occult malignancy, the presence of a genetically different cell population within the same host distorts the DNA analysis. With respect to pregnancy, the total cell-free DNA in maternal serum is analyzed with massively parallel sequencing or single-nucleotide polymorphism-based screening to estimate the risk of fetal aneuploidies. An unintended consequence of the NIPT, however, is the incidental diagnosis of somatic genetic abnormalities, including maternal aneuploidies and occult malignancy [2-6].

Pregnancy presents a unique opportunity for health screening, and to date, there are several reports of occult maternal cancers, initially suspected with routine NIPT during pregnancy [6-9], in otherwise asymptomatic patients. Here, we describe two such cases' outcomes and unique ethical considerations.

## Case presentation

Case 1

A 33-year-old gravida 5, para 3013 woman was initially referred to our maternal-fetal medicine clinic at 13 weeks, five days gestation due to abnormal NIPT results indicating an "atypical finding - outside of the scope of the test, including but not limited to fetal mosaicism, fetal chromosomal abnormality, maternal chromosomal abnormality, or normal variation". The patient had a past medical history of migraine headaches, renal calculi, and diverticulitis. After genetic counseling to discuss options for diagnostic testing, the patient opted for an amniocentesis, which was performed at 16 weeks gestation. Fluorescence in situ hybridization showed a normal fetal karyotype. The fetal anatomic survey at 20 weeks gestation was normal. Abnormal NIPT was attributed to possible placental mosaicism.

Between 25 and 27 weeks gestation, the patient was seen and evaluated at the emergency department (ED) on three separate occasions for migraines. A head computed tomography (CT) obtained at her third ED visit showed equivocal findings of low intracranial pressure without evidence of acute intracranial injury. She was discharged home in stable condition after the complete resolution of her headache. The patient was normotensive at each ED visit. At 27 weeks, three days gestation, she again presented to the ED with severe headache and sustained severe range elevated blood pressures. Treatment with 20 mg of intravenous labetalol was initiated for suspected preeclampsia with severe features. The patient was admitted for evaluation. Preeclampsia workup was otherwise negative. A magnetic resonance imaging (MRI) of the brain and spine to evaluate the etiology of her headache showed evidence of intracranial hypotension as well as cervical spine nerve root sheath diverticula suggestive of possible cerebrospinal fluid (CSF) leak. Conservative management of spontaneous CSF leak was recommended after neurosurgery and neurology consultation but without clinical improvement. A lumbar blood patch was then performed, significantly improving her headache. The patient remained normotensive for the remainder of the admission with no signs of preeclampsia. Prenatal ultrasound (US) demonstrated a fetus with an estimated fetal weight appropriate for gestational age. The patient was discharged home in stable condition with instructions for close outpatient antepartum surveillance.

**Figure 1:**
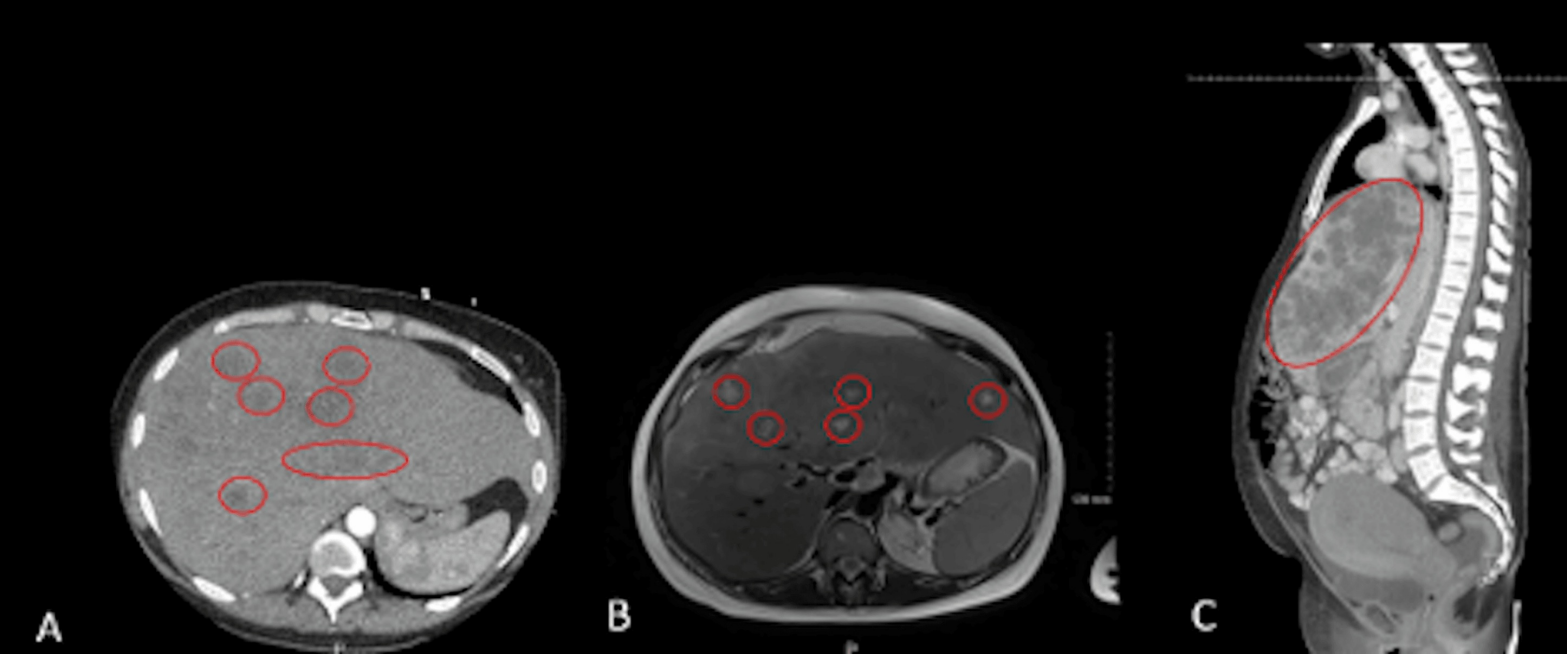
A: CT of the thorax showing multiple nonspecific hypodense liver lesions. B: Abdominal MRI and MR cholangiopancreatography showing abnormally enlarged liver with edema and inflammation as well as multiple liver lesions. C: Postpartum CT of the abdomen/pelvis showing extensive tumor involvement of the liver. CT: computed tomography, MRI: magnetic resonance imaging, MR: magnetic resonance

At 28 weeks, six days, the patient presented to the ED with new severe, persistent right upper quadrant (RUQ) abdominal pain, headache, and chest discomfort. On initial arrival, vital signs were stable, and her physical examination demonstrated RUQ tenderness. A RUQ US showed normal gallbladder but with new hepatomegaly. CT thoracic angiography was negative for pulmonary embolism but showed multiple nonspecific hypodense liver lesions (Figure 1A). Abdominal MRI and MR cholangiopancreatography showed an abnormally enlarged liver with edema and inflammation, as well as numerous liver lesions (Figure 1B). Hemoglobin was 10.2 g/dL, white blood cell count was 176,500/ µL, platelet count was 471,000/ µL, urine protein-to-creatinine ratio was 0.33, aspartate aminotransferase level (AST) was 47 IU/L, alanine aminotransferase (ALT) was 16 IU/L, alkaline phosphatase was 385 IU/L, lipase was 12 U/L, and lactic acid was 2.2 mmol/L. The patient had a negative hepatitis panel. Additionally, gamma-glutamyl transferase (GTT), carcinoembryonic antigen (CEA), and alpha-fetoprotein (AFP) were markedly elevated.

Since the MRI was done without contrast due to pregnancy, it was difficult to establish a differential diagnosis for the liver lesions: infections versus malignancy. The patient was started on broad-spectrum antibiotics while awaiting further infectious workup due to suspected liver abscesses. Additional workup, including bacterial and fungal cultures and *Histoplasma*, *Bartonella*, Epstein-Barr virus (EBV), and human immunodeficiency virus (HIV) antibodies, were all negative. The patient then underwent a liver biopsy, which showed adenocarcinoma of likely gastrointestinal origin. In the following one to two weeks, the patient clinically deteriorated with worsening abdominal pain, requiring treatment with narcotics and new supplemental oxygen requirements. The patient had previously received betamethasone for fetal lung maturity on a prior admission, and a rescue course was given during her re-admission. Delivery was recommended due to worsening maternal clinical status. She had an uncomplicated vaginal delivery at 30 weeks, one day. Postpartum CT of the abdomen/pelvis showed extensive tumor involvement of the liver (Figure 1C). A liver core biopsy was performed with a final diagnosis of stage IIIB intrahepatic cholangiocarcinoma. Chemotherapy was initiated in the early postpartum period. The patient died 23 months later.

Case 2

A 30-year-old nulliparous woman had a non-reportable NIPT result at 11 weeks gestation due to insufficient fetal DNA fraction. NIPT was repeated at 16 weeks gestation, and the result indicated an "atypical finding of likely maternal origin". After genetic consultation, the patient was enrolled in the National Institute of Health Incidental Detection of Maternal Neoplasia Through Non-Invasive Cell-Free DNA Analysis (NIH IDENTIFY) study (ClinicalTrials.gov Identifier: NCT04049604) and underwent a whole-body MRI at 25 weeks gestation, identifying a 5.5 cm right adrenal mass. A CT-guided percutaneous biopsy confirmed a diagnosis of right adrenal carcinoma with metastasis to the retroperitoneal lymph nodes. Disease progression was noted at 29 weeks gestation with a significant increase in the size of the adrenal mass to 7.7 x 6.5 x 7.9 cm with downward displacement of the right kidney and compression of the posterior inferior vena cava and liver, resulting in elevated liver function tests. Delivery by cesarean section was recommended at 30 weeks gestation with subsequent open right adrenalectomy with retroperitoneal and vena cava lymphadenectomy the next day. The patient had a final diagnosis of stage III high-grade adrenocortical carcinoma. Adjuvant systemic chemotherapy was initiated eight weeks postpartum. The patient developed malignant ascites requiring weekly paracentesis, pleural effusion, and electrolyte abnormalities. She died eight months later.

Table 1 presents the clinical presentation and outcomes of cases 1 and 2.

**Table 1:** Clinical presentation and maternal outcomes of discordant NIPT results NIPT: noninvasive prenatal test, CT: computed tomography, CTA: computed tomography angiography, MRI: magnetic resonance imaging, MRCP: magnetic resonance cholangiopancreatography

Case	NIPT result	Clinical presentation	Diagnostic tests	Final diagnosis	Outcomes
1	Atypical finding outside of the scope of the test	Nonspecific symptoms from 25 weeks gestation	Amniocentesis, head CT, CTA of the thorax, abdominal MRI and MRCP, liver biopsy, CT of the abdomen/pelvis	Stager III intrahepatic cholangiocarcinoma	Induction of labor with vaginal delivery at 30 weeks, one day gestation due to worsening maternal condition; maternal death 23 months postpartum
2	Atypical finding likely of maternal origin	Initially asymptomatic; disease progression was noted on imaging surveillance	Whole-body MRI, percutaneous CT-guided biopsy of the right adrenal gland	Stage III high-grade adrenocortical carcinoma	Cesarean delivery at 30 weeks gestation due to worsening maternal condition; maternal death eight months postpartum

## Discussion

The incidence of cancer during pregnancy is estimated to be between one in 1,000-2,000 pregnancies [10], with breast cancer being the most common malignancy diagnosed during pregnancy [11]. Cancer diagnosis and management during pregnancy presents unique ethical implications related to maternal and fetal care. Some of these ethical considerations include the potential need for iatrogenic preterm delivery or termination of pregnancy to initiate cancer treatment, as well as the possible effects of cancer chemotherapy or surgery during pregnancy [12]. The cases presented above also highlight a new, rapidly evolving ethical dilemma of disclosing abnormal NIPT results that suggest incidental cancer detection in otherwise asymptomatic patients.

Rapidly dividing apoptotic tumor cells spill tumor DNA into the maternal circulation, which can be detected by cell-free DNA sequencing. Based on this principle, the NIPT is not recommended in pregnant patients with a known cancer diagnosis [13] as this screening test is unable to distinguish between the different sources of foreign DNA (placenta or maternal tumor). However, there are multiple reports of occult maternal malignancies in otherwise asymptomatic patients initially suspected based on NIPT during pregnancy [6-9].

The TRIDENT-2 is a nationwide study that evaluated the implementation of NIPT for aneuploidy in the Netherlands for first-trimester aneuploidy screening [14]. Study participants underwent whole genome or targeted screening for common fetal aneuploidies (trisomy 21, 18, and 13). A follow-up retrospective review of the study population with malignancy-suspicious NIPT showed that most cases (94.3%) were identified with whole genome sequencing compared to the targeted sequencing [4]. All cases of malignancy-suspicious NIPT underwent an invasive diagnostic prenatal evaluation to confirm discordant NIPT results. Subsequently, patients with discordant NIPT underwent additional diagnostic testing and imaging based on symptoms and the nature of the underlying malignancy suspected. Biopsy-proven malignancy was confirmed in 33.3% (n=16) of the 48 patients with malignancy-suspicious NIPT cases. The most common finding associated with confirmed malignancy was multiple (≥2) chromosomal aberrations, noted in 69.6% of the participants. Of note, most cancers diagnosed in this population were of hematologic origin (Hodgkin's lymphoma, non-Hodgkin's lymphoma, and acute myeloid leukemia), followed by breast cancer and colorectal cancer. The authors hypothesized that hematologic cancers, compared to solid tumors, may be more easily detectable by NIPT due to direct access to circulating maternal plasma [4].

Several technical (specimen handling or processing) and biological factors (e.g., low fetal DNA fraction) may contribute to abnormal NIPT results. Occult malignancy accounts for a small fraction of potential causes of atypical NIPT results. Additionally, the current evidence in the literature suggests that most cancers are likely not detectable with NIPT, as only 0.01% of the total TRIDENT 2 population were ultimately diagnosed with premalignant or malignant tumors [4,14]. This finding has been replicated in a Belgian population [15].

The utility of NIPT for cancer screening is yet to be established due to many challenges. In the setting of occult maternal malignancy, the paucity of cases detectable in the early stages is an important limiting step in establishing the clinical utility of NIPT for cancer screening. Additionally, invasive and noninvasive diagnostic testing in patients with NIPT suspicious but not diagnostic of occult malignancy has significant cost and ethical implications. In addition, the uncertain clinical significance of discordant NIPT results presents a counseling conundrum to clinicians and geneticists while also potentially increasing patients' anxiety. Controlling for age, stage, and subtype of disease and treatment, the prognosis for breast, gynecological cancers, and Hodgkin's lymphoma is comparable in cases initially diagnosed in pregnancy when compared to non-pregnant patients [10]. However, there is limited data for more aggressive cancers such as cholangiocarcinoma or metastatic adrenal cancer, as presented above. Moreover, it is concerning that when multiple chromosomal aberrations are found, the risk of a confirmed malignancy is considerably high, as reported in some studies [5].

The IDENTIFY study (ClinicalTrials.gov Identifier: NCT04049604) is a prospective observational cohort study aiming to evaluate the natural history of maternal neoplasia in patients with prenatal screening suspicious for underlying maternal cancer. All eligible patients undergo an initial evaluation, whole-body MRI, and emotional well-being assessment, with subsequent referral based on MRI findings. Depending on the etiology, stage, and prognosis, cancer diagnosis during pregnancy presents unique ethical challenges related to maternal and fetal well-being.

Given the limited standardized clinical guidelines, a multidisciplinary approach has been proposed for all pregnant patients with atypical NIPT results [10]. All pregnant patients with "atypical or non-reportable" NIPT results should be referred to a maternal-fetal medicine specialist and genetic counselor for evaluation, including a history and physical examination. Patients are offered prenatal diagnostic testing (chorionic villus sampling or amniocentesis, depending on the gestational age), and those diagnosed with fetal aneuploidy are managed accordingly. For patients with a normal fetal karyotype or fetal anatomic survey (discordant NIPT results), a referral to the NIH IDENTIFY study (ClinicalTrials.gov Identifier: NCT04049604) is encouraged. Based on the initial evaluation, additional subspecialty referral should be pursued for further workup, biopsy, and cancer staging as indicated. All pregnant patients with biopsy-proven malignancy should be followed closely by a maternal-fetal medicine specialist for antenatal care. Delivery timing will be determined based on the underlying cancer diagnoses, the need for chemotherapy, and maternal-fetal status. Cancer diagnosis and treatment during the reproductive age group or pregnancy has potential implications on future fertility. As a result, all patients with biopsy-proven cancer may also be offered a referral to a reproductive endocrinologist for fertility preservation counseling.

## Conclusions

Noninvasive prenatal testing is not validated for cancer screening or diagnosis in pregnant and non-pregnant people. Although rare, there have been several reports of incidental diagnosis of underlying maternal cancer in otherwise asymptomatic patients. A multidisciplinary approach should be considered when evaluating cases of atypical NIPT results. Patients should be referred to a prenatal geneticist and maternal-fetal medicine expert for genetic counseling, diagnostic genetic testing, and fetal anatomic survey, as indicated. Patients with discordant NIPT results should be monitored closely during pregnancy and postpartum, and nonspecific or atypical symptoms should be evaluated thoroughly.
